# Supplementary material for: A library of MiMICs allows tagging of genes and reversible, spatial and temporal knockdown of proteins in Drosophila
Source: eLife. 2015 Mar 31;4:e05338. doi: 10.7554/eLife.05338 (PMC4379497; doi:10.7554/eLife.05338)
Supplement: Figure 1—source data 2. — DOI: http://dx.doi.org/10.7554/eLife.05338.005 [file elife05338s002.docx]

**Figure 1–Source data 2 :** List of fly strains used in the study

| **Fly base number** | **Genotype** |
| --- | --- |
| FBst0025688 | *w1118; Df(3L)BSC613/TM6C, cu1 Sb1* |
| FBst0007538 | *w1118; Df(2R)Exel6056, P{XP-U}Exel6056/CyO* |
| FBst0007838 | *w1118; Df(2L)Exel7068/CyO* |
| FBst0007541 | *w1118; Df(2R)Exel6059, P{XP-U}Exel6059/CyO* |
| FBst0012144 | *y[1] w[*]; P{w[+mC]=lacW}vib[j5A6]/TM6B, Tb[+]* |
| FBst0002363 | *Df(3R)crb87-5, st1 e1/TM3, Ser1* |
| FBst0023663 | *w1118; Df(2L)BSC278/CyO* |
| FBst0007696 | *w1118; Df(3R)Exel6218, P{XP-U}Exel6218/TM6B, Tb1* |
| FBst0053782 | *y[1] w[*]; Mi{y[+mDint2]=MIC}unk[MI09783]/TM3, Sb[1] Ser[1]* |
| FBst0002992 | *Df(3L)Ly, Df(3L)BK10, ru1 sensLy-1 red1 cv-c1 Sbsbd-1 sr1 e1/TM3, Sb1* |
| FBst0007566 | *w1118; Df(3L)Exel6087, P{XP-U}Exel6087/TM6B, Tb1* |
| FBst0007744 | *w1118; Df(2L)Exel6277, P{XP-U}Exel6277/CyO* |
| FBst0026179 | *Trpml^6^ red^1^ e^4^/TM6B, Sb^1^ Tb^1^ ca^1^* |
| FBst0001687 | *Rdl^1^/TM3, Sb^1^* |
| FBst0006756 | *Df(3R)BSC24, st1 ca1/TM3, Ser1* |
| FBst0008886 | *Df(2R)cos-2, cn1 bw1 sp1/CyO* |
| FBst0009289 | *w1118; Df(3R)BSC124/TM6B, Tb1* |
| FBst0007548 | *w1118; Df(2R)Exel6066, P{XP-U}Exel6066/CyO* |
| FBst0004513 | *w[*]; Eip63E[81]/TM6B, Tb[1]* |
| FBst0007610 | *w1118; Df(3L)Exel6131, P{XP-U}Exel6131/TM6B, Tb1* |
| FBst0007413 | *Df(3R)BSC43, st1 ca1/TM2, pp* |
| FBst0007745 | *w1118; Df(3L)Exel6279, P{XP-U}Exel6279/TM6B, Tb1* |
| FBst0007079 | *Df(3L)BSC35, rhove-1 e1/TM3, P{Ubx-lacZ.w+}TM3, Sb1* |
| FBst0007734 | *w1118; Df(3R)Exel6267, P{XP-U}Exel6267/TM6B, Tb1* |
| FBst0009845 | *y1 w*; P{UAST-YFP.Rab9Fb.S21N}Gαo01/CyO* |
| FBst0000997 | *Df(3L)AC1, rnroe-1 pp/TM3, Sb1* |
| FBst0019819 | *y^1^ w^67c23^; P{EPgy2}bon^EY01763^/TM3, Sb^1^ Ser^1^* |
| FBst0007521 | *w1118; Df(2L)Exel6038, P{XP-U}Exel6038/CyO* |
| FBst0005425 | *w*; Df(2R)12/CyO* |
| FBst0024325 | *w1118; Df(2R)BSC327/CyO* |
| FBst0010490 | *y1 w67c23; P{lacW}edk01102/CyO* |
| FBst0007855 | *w1118; Df(2L)Exel7081/CyO* |
| FBst0002785 | *cn1 fas1 bw1 sp1/CyO* |
| FBst0031020 | *y1 w*; Mi{MIC}simaMI00480/TM6B, Tb1* |
| FBst0007516 | *w1118; Df(2L)Exel6033, P{XP-U}Exel6033/CyO* |
| FBst0007666 | *w1118; Df(3R)Exel6187, P{XP-U}Exel6187/TM6B, Tb1* |
| FBst0055425 | *y1 w*; Mi{MIC}CG31272MI05954* |
| FBst0007497 | *w1118; Df(2L)Exel6011, P{XP-U}Exel6011/CyO* |
| FBst0025398 | *w*; Df(3L)469-6/TM6B, Tb1* |
| FBst0003554 | *Abl1 kar1 red1 e1/TM6B, Tb1* |
| FBst0010900 | *y1 w*; P{lacW}hdcB4-3-20/TM3, Sb1* |
| FBst0012326 | *cn1 P{PZ}for06860/CyO; ry506* |
| FBst0007516 | *w1118; Df(2L)Exel6033, P{XP-U}Exel6033/CyO* |
| FBst0000258 | *Df(2R)Np3, bw1/CyO* |
| FBst0034300 | *y[1] w[*]; Mi{y[+mDint2]=MIC}fred[MI02235]/SM6a* |
| FBst0007646 | *Df(3R)Exel6167* |
| FBst0007294 | *Df(3L)4231/TM3, P{GAL4-Kr.C}DC2, P{UAS-GFP.S65T}DC10, Sb1* |
| FBst0024630 | *y1 w*; unc-104P350/CyO* |
| FBst0011097 | *y1 w67c23; P{lacW}Sema-1ak13702/CyO* |
| FBst0007692 | *w1118; Df(3R)Exel6214, P{XP-U}Exel6214/TM6B, Tb1* |
| FBst0007993 | *w1118; Df(3R)Exel8178/TM6B, Tb1* |
| FBst0006075 | *Df(2L)b75/CyO* |
| FBst00042114 | *y[1] w[*]; Mi{y[+mDint2]=MIC}Cyp4p3[MI05774] hig[MI05774]/SM6a* |
| FBst0007528 | *spir[1] cn[1] bw[1]/CyO, l(2)DTS513[1]* |
| FBst0006344 | *Df(2L)ast5/SM1* |
| FBst0002604 | *Df(2R)Px2/CyO, P{sevRas1.V12}FK1* |
| FBst0007602 | *w1118; Df(3R)Exel6193, P{XP-U}Exel6193/TM6B, Tb1* |
| FBst0002998 | *Df(3L)81k19/TM6B, Tb1* |
| FBst0007294 | *Df(3L)4231/TM3, P{GAL4-Kr.C}DC2, P{UAS-GFP.S65T}DC10, Sb1* |
| FBst0003360 | *Df(3R)4SCB/TM3, Sb^1^ Ser^1^* |
| FBst0007783 | *w1118; Df(2L)Exel7011/CyO* |
| FBst0006764 | *y1 w*/Dp(1;Y)y+; Df(2R)XTE-11/CyO* |
| FBst0024983 | *w1118; Df(3R)BSC479/TM6C, Sb1 cu1* |
| FBst0024924 | *w1118; Df(3L)BSC420/TM6C, Sb1 cu1* |
| FBst0002583 | *Df(2L)cact-255rv64, cactchif64/CyO; ry506* |
| FBst0003548 | *Df(2L)al, dsal/In(2L)Cy, Cy1* |
| FBst0007554 | *w1118; Df(2R)Exel6072, P{XP-U}Exel6072/CyO* |
| FBst0000823 | *Df(3R)D605/TM3, Sb1 Ser1* |
| FBst0007831 | *w1118; Df(2L)Exel7063/CyO* |
| FBst0007663 | *w1118; Df(3R)Exel6184, P{XP-U}Exel6184/TM6B, Tb1* |
| FBst0032699 | *y[1] w[*]; Mi{y[+mDint2]=MIC}foxo[MI00786]/TM3, Sb[1] Ser[1]* |
| FBst0004371 | *ru[1] h[1] nrm[48] ry[506] sr[1] e[s] ca[1]/TM6B, P{w[+mC]=iab-2(1.7)lacZ}6B, Tb[+]* |
| FBst0025060 | *Df(1)BSC532, w1118/FM7h/Dp(2;Y)G, P{hs-hid}Y* |
| FBst0007940 | *w1118; Df(3L)Exel9006/TM6B, Tb1* |
| FBst0027504 | *Df(3R)HTRI/TM3, P{Thb8-lacZ}WD1, Sb1 Ser1* |
| FBst0034237 | *y[1] w[*]; Mi{y[+mDint2]=MIC}dpr12[MI01695]* |
| FBst0040738 | *y[1] w[*]; Mi{y[+mDint2]=MIC}dpr11[MI01743]* |
| FBst0041434 | *y^1^ w^*^; Mi{MIC}nAChRα5^MI05549^* |
| FBst0003467 | *Df(2R)AA21, c1 px1 sp1/SM1* |
| FBst0007871 | *w1118; Df(2R)Exel8057/CyO* |
| FBst0012088 | *w1118; P{lacW}Trls2325/TM3, Sb1* |
| FBst0006964 | *w[1118]; Df(3R)ED6025, P{w[+mW.Scer\FRT.hs3]=3'.RS5+3.3'}ED6025/TM6C, cu[1] Sb[1]* |
| FBst0001866 | *Df(3R)dsx15/TM2* |
| FBst0007977 | *w1118; Df(3R)Exel7321/TM6B, Tb1* |
| FBst0007724 | *w1118; Df(2L)Exel6256, P{XP-U}Exel6256/CyO* |
| FBst0007652 | *w1118; Df(3R)Exel6173, P{XP-U}Exel6173/TM6B, Tb1* |
| FBst0009078 | *w1118; Df(3R)ED5438, P{3'.RS5+3.3'}ED5438/TM6C, cu1 Sb1* |
| FBst0008825 | *Df(2L)A92, nub1 b1 pr1/CyO* |
| FBst0002363 | *Df(3R)crb87-5, st1 e1/TM3, Ser1* |
| FBst0006957 | *Df(2R)11, cn1/CyO, amosRoi-1* |
| FBst0007542 | *w1118; Df(2R)Exel6060, P{XP-U}Exel6060/CyO* |
| FBst0042316 | *y1 w*; Mi{MIC}unc-5MI05371* |
| FBst0007500 | *w1118; Df(2L)Exel6014, P{XP-U}Exel6014/CyO* |
| FBst0041424 | *y^1^ w^*^; Mi{MIC}nAChRα5^MI05549^* |
| FBst0034444 | *y^1^ w^*^; Mi{MIC}SPoCk^MI01547^* |
| FBst0007674 | *w1118; Df(3R)Exel6191, P{XP-U}Exel6191/TM6B, Tb1* |
| FBst0008000 | *w1118; Df(2L)Exel6006, P{XP-U}Exel6006/CyO* |
| FBst0007584 | *w1118; Df(3L)Exel6105, P{XP-U}Exel6105/TM6B, Tb1* |
| FBst0002363 | *Df(3R)crb87-5, st1 e1/TM3, Ser1* |
| FBst0030649 | *y[1] w[*]; Mi{y[+mDint2]=MIC}Ac76E[MI00178]/TM6B, Tb[1]* |
| FBst0044667 | *y^1^ w^*^; Mi{MIC}Roc2^MI01663^* |
| FBst0037958 | *y^1^ w^*^; Mi{MIC}dpr6^MI04582^* |
| FBst0007789 | *w1118; Df(2L)Exel7018/CyO* |
| FBst0036004 | *y[1] w[*]; Mi{y[+mDint2]=MIC}SerT[MI02578]* |
| FBst0006344 | *Df(2L)ast5/SM1* |
| FBst0007623 | *w1118; Df(3R)Exel6144, P{XP-U}Exel6144/TM6B, Tb1* |
| FBst0000416 | *w1118; Df(3L)GN19/TM3, ry* su(Hw)2 Sb1* |
| FBst0043905 | *y^1^ w^*^; Mi{MIC}sif^MI07526^/TM3, Sb^1^ Ser^1^* |
| FBst0007741 | *w1118; Df(3R)Exel6274, P{XP-U}Exel6274/TM6B, Tb1* |
| FBst0043558 | *y[1] w[*]; Mi{y[+mDint2]=MIC}Pxn[MI04044]/TM3, Sb[1] Ser[1]* |
| FBst0007646 | *w1118; Df(3R)Exel6167, P{XP-U}Exel6167/TM6B, Tb1* |
| FBst0024384 | *w1118; Df(2R)BSC360/CyO* |
| FBst0007692 | *w1118; Df(3R)Exel6214, P{XP-U}Exel6214/TM6B, Tb1* |
| FBst0007752 | *w1118; Df(3R)Exel6288, P{XP-U}Exel6288/TM6B, Tb1* |
| FBst0024911 | *w1118; Df(2L)BSC407/CyO* |
| FBst0030644 | *y[1] w[*]; Mi{y[+mDint2]=MIC}Gp210[MI00167]/CyO* |
| FBst0053790 | *y^1^ w^*^; Mi{MIC}Meltrin^MI09878^/TM3, Sb^1^ Ser^1^* |
| FBst0032816 | *y^1^ w^*^; Mi{MIC}Hmx^MI02025^/TM3, Sb^1^ Ser^1^* |
| FBst0004431 | *Df(3R)DG2/TM2, red1* |
| FBst0025689 | *w1118; Df(3L)BSC614/TM6C, cu1 Sb1* |
| FBst0036163 | *y^1^ w^*^; Mi{MIC}Pk92B^MI02915^/TM3, Sb^1^ Ser^1^* |
| FBst000442 | *Df(2R)CX1, wg12 b1 pr1/SM1* |
| FBst038553 | *y^1^ w^*^; Mi{MIC}heph^MI03238^/TM3, Sb^1^ Ser^1^* |
| FBst038131 | *y^1^ w^*^; Mi{MIC}sm^MI04033^/SM6a* |
| FBst0004972 | *cn^1^ cha^HB^ bw^1^/CyO, l(2)DTS513^1^* |
| FBst0036529 | *w1118; Df(3R)BSC677, P+PBac{XP3.WH3}BSC677/TM6C, Sb1 cu1* |
| FBst0002992 | *Df(3L)Ly, Df(3L)BK10, ru1 sensLy-1 red1 cv-c1 Sbsbd-1 sr1 e1/TM3, Sb1* |
| FBst0007657 | *w1118; Df(3R)Exel6178, P{XP-U}Exel6178/TM6B, Tb1* |
| FBst0024982 | *w1118; Df(3R)BSC478/TM6C, Sb1 cu1* |
| FBst0006089 | *Df(2L)TE37C-7/CyO* |
| FBst0007490 | *w1118; Df(2L)Exel6003, P{XP-U}Exel6003/CyO* |
| FBst0007692 | *w1118; Df(3R)Exel6214, P{XP-U}Exel6214/TM6B, Tb1* |
| FBst0041555 | *y^1^ sc^*^ v^1^; P{VALIUM20-EGFP.shRNA.1}attP40* |
| FBst0041560 | *y^1^ sc^*^ v^1^; P{VALIUM20-EGFP.shRNA.3}attP2* |
| FBst0038422 | *y1 w*; P{UAS-Nslmb-vhhGFP4}2* |
| FBst0038421 | *w*; P{UAS-Nslmb-vhhGFP4}3* |
